# Single-incision laparoscopic surgery: An overview

**DOI:** 10.4103/0972-9941.72354

**Published:** 2011

**Authors:** Tehemton E Udwadia

**Affiliations:** Department of Minimal Access Surgery, P. D. Hinduja National Hospital, Veer Savarkar Marg, Mahim, Mumbai, India. E-mail: t_udwadia@hotmail.com

Over the last two decades, conventional multi-port Minimal Access Surgery (MAS) has established itself as the gold standard for almost all abdominal surgical procedures. The procedure provides safety, ease, undisputed patient benefit at a cost acceptable to the healthcare system by surgeons from several specialties all over the world, in large hospitals as well as underprivileged rural areas. MAS has effectively addressed the patients’ right to less scarring, trauma (both of access and intra-abdominal manipulation), medication, pain, hospitalization, and early return to family and work.

The only truth in surgery is change. Reducing scars and the insult of surgical trauma has become a vital end point of all surgical assessment and endeavour. In the pursuit of further reduction in scarring and the trauma of multi-port MAS, surgeons and the instrument industry have combined their ingenuity and expertise to promote two new approaches for MAS – Natural Orifice Trans-luminal Endoscopic Surgery (NOTES) and Single-Incision Laparoscopic Surgery (SILS). NOTES leaves no skin scars, but requires entry into the peritoneal cavity with the use of flexible endoscopes by perforation of a hollow viscus, the stomach oesophagus, colon, and bladder. It further necessitates a collage of various types of endoscopes, instrumentation, and techniques that are largely alien to surgeons, to many of whom making and maintaining for the duration of the procedure a viscus perforation may seem repugnant. Historically, surgery has always been performed by two hands of the surgeon working in synergy by hand contact in open surgery, which is now done by the long instruments in MAS. SILS performs the same procedure as multi-port MAS through only one incision at the umbilicus in the expectation of reducing scarring and surgical trauma. Hence, SILS could perhaps be accepted as the progression of multi-port MAS by surgeons, and could be more in consonance with their psyche and technology than NOTES.

This is confirmed by the rapid spread of SILS worldwide in just two or three years. The number of surgeons who enthusiastically practice and advocate SILS is increasing rapidly, and the instrument industry has a strong thrust towards increasing the safety and evolving the technology for SILS. SILS has been performed in the full spectrum of all abdominal surgery, gallbladder, appendix, colon, spleen, adrenals, and kidney, with results reported comparable to multi-port MAS. Hence, this issue of *Journal of Minimal Access Surgery (JMAS)* will focus only on SILS.

Having extolled the safety, comparable morbidity and good results of SILS, one needs to pause and evaluate SILS in its totality. The basis of safe and smooth multi-port MAS is the ability to secure perfect triangulation at the tips of the hand instruments, just like the hands of the surgeon during open surgery, which ensured that conventional MAS was accepted as the logical progression of open surgery. The design and complexity of several ports advocated, size and number of incisions at the umbilicus highlight just some of the complexities inherent to SILS. Safe and smooth MAS necessitates the triangulation of the hand instrument tips. With parallel trocars and telescope inserted just a few millimeters apart, it requires ingenuity of instrumentation and technique to achieve a degree of triangulation also slightly comparable with the perfect triangulation of multi-port surgery. A vast array of such hand instruments has evolved, all of which are curved coaxial instruments with outward deviation proximally, each generation of which gives greater ease of performance, yet becomes more complex and more expensive.

The raison d’être of NOTES and SILS is less scarring, less surgical trauma. NOTES leaves no scars, while SILS leaves at least a 1.5-cm – 2-cm scar at the umbilicus. With a marked improvement in design, diversity, and durability of 3-mm hand instruments and 3-mm optics, the multi-port MAS is increasingly being performed with 3-mm punctures, reducing scarring, and trauma. Industry and media hype may ensure that patients demand procedures with minimal or no scar. However, surgeons appreciate that there is far more to assessing any procedure than mere size or absence of scar. In general surgery, the degree of surgical trauma (and scarring) was simple to quantify and compare – from a supra-radical, radical, modified radical mastectomy to breast-conserving surgery left no doubt about decreasing trauma. How accurately can one quantify the degree of surgical trauma even with the most sophisticated methods of evaluation, cell response, metabolic, inflammatory, biochemical, and others in current MAS? NOTES does not include a skin incision and claims minimal surgical trauma. Is a flexible endoscope positioned in situ for one to three hours, penetration of a hollow viscus, a rent that is kept open throughout the procedure, transmigration of pathogens into the peritoneal cavity, tissue retrieval, and final closure of the rent devoid of all surgical trauma? Is a 1-cm incision at the umbilicus and three 5-mm or 3-mm punctures in multi-port MAS a cause of infinitely greater surgical trauma than a 2-cm incision of both skin and abdominal walls at the umbilicus as in SILS? This may, perhaps, be an appropriate time to re-evaluate our methods of assessment of surgical trauma and insult, which may have subtleties and innuendos that the currently available assays may not measure, considering that the results of surgical trauma can manifest later.

It seems that the future of SILS, if left to the interests of industry, will in sequence be dependent or associated with robotics and even greater sophistication. One needs to pause and ponder whether this industry generated euphoric hubris of amazingly intricate and cripplingly expensive equipment - articulating hands, robots, simulators, data processors, computers, etc. will strangle the future of SILS, confining the procedure to isolated, richly funded ivory towers of excellence. Healthcare costs are a major factor for the economical crisis in several countries, resulting in rapid deterioration of healthcare delivery all over the world. Surgery is a humanitarian science whose benefit should reach all people in all places. Manufacturers may continue with their research and advances, but they need to be a little restrained to ensure that affordability and spread worldwide is not compromised. However, there will always be hope for the growth and spread of SILS with simple, affordable solutions. Percutaneous retraction sutures on gallbladder or stomach, a method for one-handed intracorporeal knot tying, the “re-invention” of the old Storz operative laparoscope, which in the 1960s-1980s played a major role in the tubal sterilisation population control programmes of a number of developing countries, are a few of the affordable innovations born out of financial stringency that will help keep SILS on an even keel. For recalling the statement of two surgeons a few millennia apart, Sushruta, who in 800 BC invented and used scores of surgical instruments, taught his students never to stop designing new instruments, yet never to forget that the best surgical instrument is the surgeon’s hand. Closer to our times, Phillippe Mouret, who set the avalanche of MAS rolling, repeatedly emphasised that it is for the surgeon, and not the manufacturer, to decide which instruments the surgeon will use.

SILS stands where multi-port MAS stood in 1988. It did have few staunch advocates but was viewed then at best with indifference by some, and more often with skepticism or hostility by the rest. Conventional MAS proved to become the gold standard by virtue of its patient benefit, safety, wide applicability, acceptance, and performance in most parts of the world. The method, which was under trial, had come to stay. Today, SILS is a method under trial, in an endeavour to introduce a more patient-beneficial advance over multi-port MAS. Time and pragmatic evaluation will tell whether this method is only something new or is truly better in terms of patient benefit; safety; low morbidity, both early and late; ease of performance by all surgeons conversant with MAS; cost-effectiveness comparable to conventional MAS, thus ensuring its widespread application. If proved better on all counts, SILS will, in time, replace multi-port MAS as the next gold standard of MAS.

It is to explore the cutting edge of this advance that the Editorial Board, with the unstinted effort of one of the Editors, Dr Deepraj S Bhandarkar, has brought out this special issue on SILS. The *JMAS* is honoured and privileged to have one of its Editors, Professor Sir Alfred Cuschieri, pen his erudite, balanced, pragmatic yet futuristic Editorial to this special issue of *JMAS*.

